# The Organisation in North Lambeth

**Published:** 1918-01-26

**Authors:** 


### PREPARING FOR AIR RAIDS.

### The Organisation in North Lambeth.

Mr. Frank Briant, J.P., L.C.C., the Chairman of the Lambeth Board of Guardians, has achieved a remarkable success in organising what is known as the North Lambeth and Kennington Air-raid Volunteer Corps for the supervision of the air-raid shelters in the district and for rousing the people in case of a warning being received. This is said to be the first organised attempt in London to deal with the needs of women and children in air-raid shelters. After one of the first raids Mr. Briant realised that it was essential for the safety of the residents that at each shelter there should be men specially stationed to keep order, and fully-qualified women to attend to the women and children. He called a meeting of those ready to assist, when he received promises from 160 men and women, and within a few weeks the number of voluntary workers had swelled to over 900.

There are twenty-six shelters in the district, irrespective of the Tubes, consisting of arches, factory and warehouse basements, L.C.C. schools, and churches and chapels, and in these from 18,000 to 20,000 persons assemble for shelter. The organisation, under the direct control of Mr. Briant, has its headquarters at Alford House, Lambeth Walk, where arrangements have been made to lodge dishoused men and boys, whilst, on the opposite side of the street, at Pelham Hall, the women and children are sheltered when rendered homeless.

### The Nursing Arrangements.

Mr. Briant's first object was to secure at each shelter the services of two qualified women with either nursing or V.A.D. experience to attend to women and children in cases of emergency. Where he has been unable up to the present to secure these, he has obtained the best substitute possible, many women of strong nerve and capable experience agreeing to assist. These are also helped by a number of girls affiliated either to the Red Cross or the V.A.D., who are equipped with knapsacks containing restoratives and first-aid appliances. In more serious cases a trained nurse is summoned, or the patient is removed to Lambeth Infirmary.

Mr. Briant followed this up by applying to the two certified midwives living in the district, asking them if they would be willing to assist in maternity cases, of which, unfortunately, three had occurred at the shelters during air raids. These ladies at once agreed willingly to place their services at his disposal. At each shelter Mr. Briant has also arranged for four men to be in attendance to keep order.

### The Street Patrols.

The second part of Mr. Briant's work was the organisation of street patrols. This has been so perfected that every narrow street, court, and alley has its patrol. The men, all from the working classes, wear special badges, and carry membership cards, signed by Mr. Briant, which are recognised by the police, with whom Mr. Briant's force works in thorough agreement.

The duties of the patrol in case of an air-raid warning are at once to call the helpers—men and women—and the rest of their patrol, to arouse the women and children in their street, and to assist in getting them to the shelters. The men are on duty two hours at a time from early in the evening until six o'clock next morning, and a number of shelters have been placed at their disposal, where fires are provided so that if they desire they may make themselves tea or coffee.

The patrol is a voluntary organisation. The poor are ready to give their mite towards the cost of the work, or by a monetary gift to show their gratification for the services rendered them in times of distress. All money given in this way is entered by the patrols on recognised subscription lists, and is expended on the purchase of blankets, towels, restoratives, and first-aid material.

### Mr. Briant and His Work.

Such is the work Mr. Briant has done for the better protection of the inhabitants of North Lambeth and Kennington. It is a huge work, and is constantly growing, for outside the district, even from the adjoining borough of Southwark, applications are being made to him for inclusion in his organisation. Much of the success is undoubtedly due to the strong personality of Mr. Briant in the district, where for many years he has carried on a club for men and boys—a centre of philanthropic and useful work—at Alford House. He remains in the district all night when there is an air raid, and visits the various shelters to see that all his helpers are present and doing their utmost to relieve the anxiety of those seeking shelter.

The advantages of a voluntary organisation of this kind, under the control of a responsible head, are apparent. There is an antipathy amongst many people—especially amongst the poor—to register at the police stations for service, but, should there be a similar voluntary organisation in any other district, it is felt they would be ready to place their training and experience at the service of the community. Hence there is room for similar voluntary organisations in all the crowded and poor districts of London.

